# Editorial Comment to Knotted ureteral single‐J stent in a patient with ureterocutaneostomy

**DOI:** 10.1002/iju5.12364

**Published:** 2021-08-24

**Authors:** Yohei Sekino, Jun Teishima

**Affiliations:** ^1^ Department of Urology Graduate School of Biomedical and Health Sciences Hiroshima University Hiroshima Japan

Ureteral stent catheterization is a common procedure in urological practice. Although ureteral stents have undergone notable technological advancements in the past few decades, numerous side effects exist and affect the patient physically and psychologically.^1^ The knotting ureteral stent is a serious and rare complication. This case report showed that the author encountered the knotting ureteral stent during the routine exchange and successfully removed it using a flexible cystoscope.^2^

In this case report, a ureteral stent for cutaneous ureterostomy was changed every month. One paper showed that the catheter‐free rate was 70%–80% for cutaneous ureterostomy.^3^ Although it might be difficult to create the tubeless cutaneous ureterostomy because the patient had only one functional kidney, it might be good to create the tubeless cutaneous ureterostomy.

In this case report, the author changed the ureteral stent without fluoroscopy. Sometimes, experienced urologists change a ureteral stent without fluoroscopy. A recent paper regarding the techniques and tips for ureteral stent placement demonstrated that a ureteral stent is changed under fluoroscopy and retrograde pyelography.^3^ To our knowledge, there is no guidance regarding the use of fluoroscopy for ureteral stent replacement. As the author mentioned in this case report, ureteral stent replacement should be performed with imaging help.

So far, 30 reports about the knotting stent have been reported.^2^ In this case report, the author reviewed several techniques for the removal of knotting stent. This paper will help to make strategies for knotting stent.

## Conflict of interest

The authors declare no conflict of interest.
